# Efficacy, safety, and cost-effectiveness analysis of aflibercept in metastatic colorectal cancer: A rapid health technology assessment

**DOI:** 10.3389/fphar.2022.914683

**Published:** 2022-08-30

**Authors:** Pu Ge, Ning Wan, Xiao Han, Xinpei Wang, Jinzi Zhang, Xiaoyi Long, Xiaonan Wang, Ying Bian

**Affiliations:** ^1^ Institute of Chinese Medical Sciences, University of Macau, Macau, China; ^2^ State Key Laboratory of Quality Research in Chinese Medicine, University of Macau, Macau, China; ^3^ Department of Public Health and Medicinal Administration, Faculty of Health Sciences, University of Macau, Macau, China; ^4^ General Hospital of Southern Theater Command, Guangzhou, Guangdong, China; ^5^ Guangdong Branch Center, National Clinical Research Center for Geriatric Diseases (Chinese PLA General Hospital), Guangzhou, Guangdong, China; ^6^ School of Pharmaceutical Sciences, Sun Yat-Sen University, Guangzhou, China; ^7^ Institute of Health and Wellbeing, University of Glasgow, Glasgow, United Kingdom; ^8^ College of Humanities and Social Sciences, Harbin Medical University, Harbin, China; ^9^ The First Affiliated Hospital of Medical School of Zhejiang, Hangzhou, China; ^10^ School of Traditional Chinese Pharmacy, China Pharmaceutical University, Nanjing, China

**Keywords:** metastatic colorectal cancer, aflibercept, rapid health technology assessment, targeted drugs, cost-effectiveness analysis, pharmacoeconomics

## Abstract

**Background:** Metastatic colorectal cancer (mCRC) imposes a heavy tumor burden worldwide due to limited availability of therapeutic drugs. Aflibercept, a kind of recombinant protein of the anti-vascular endothelial growth factor (VEGF) family, has been approved in clinical application among mCRC patients since 2012. A comprehensive analysis of the efficacy, safety, and cost-effectiveness of aflibercept in mCRC treatment is necessary.

**Objective:** To evaluate the efficacy, safety, and cost-effectiveness of aflibercept for the treatment of mCRC in order to provide a decision-making reference for the selection of targeted drugs for second-line treatment of mCRC in Hong Kong, Macao, and Taiwan regions of China and the selection of new drugs for medical institutions in these regions.

**Methods:** A systematic retrieve on databases including PubMed, Embase, Cochrane Library, China National Knowledge Infrastructure (CNKI), Wanfang, and Weipu, as well as relevant websites and databases of health technology assessment including the National Institute of Health and Clinical Optimization, Centre for Evaluation and Communication at the University of York, and the Canadian Agency for Medicines and Health Technology, was conducted. The literature was screened according to the inclusion and exclusion criteria, and data were extracted and analyzed by two authors, while the quality of the literature was assessed.

**Results:** Finally, we included two HTA reports, 11 systematic reviews/meta-analyses, and two cost-effectiveness studies in the rapid health technology assessment. For mCRC patients receiving second-line treatment, aflibercept combined with FOLFIRI significantly increased progression-free survival (PFS) and overall survival (OS) and the objective response rate (ORR) also improved, compared with folinic acid + fluorouracil + irinotecan (FOLFIRI). In terms of safety, mCRC patients who received aflibercept combined with FOLFIRI therapy had a higher incidence of grade 3–4 adverse events than those who received FOLFIRI alone, including anti-VEGF–related adverse events (hypertension, hemorrhagic events, and proteinuria) and chemotherapy-related adverse events (diarrhea, weakness, stomatitis, hand-foot syndrome, neutropenia, and thrombocytopenia). In terms of cost-effectiveness, two economic studies conducted in the United Kingdom and Japan, respectively, found that compared with FOLFIRI, aflibercept combined with FOLFIRI had no cost-effectiveness advantage in mCRC patients receiving second-line treatment.

**Conclusion:** Compared with FOLFIRI treatment, aflibercept combined with FOLFIRI for the second-line treatment of mCRC patients has better efficacy, worse safety, and is not cost-effective. More high-quality clinical studies are required for further exploration of aflibercept’s clinical value. Medical institutions in Hong Kong, Macao, and Taiwan regions of China should be cautious when using or introducing aflibercept plus FOLFIRI as a mCRC treatment.

## 1 Introduction

Colorectal cancer, as one of the most common gastrointestinal malignancies, features high incidence, high death rate, and low cure rate and seriously threatens human health. The 2020 data showed that the incidence and mortality rate of colorectal cancer in the world, respectively, ranked third and second of all cancers, of which 1.932 million were new cases and 935,000 deaths (Sung et al., 2021). Patients with early colorectal cancer show lack of specific symptoms, and the screening for it is not commonly performed in most parts of the world (Halama and Haberkorn, 2020). All of these reasons make early diagnosis of colorectal cancer difficult; therefore, most patients are diagnosed in the middle or late stages and may even have metastatic colorectal cancer (mCRC). According to the anatomy of the splenic flexure, colorectal cancer can be divided into left colon cancer and right colon cancer, with approximate incidence of 69.6% and 30.3%, respectively (Broman et al., 2019). About 50% of patients with colorectal cancer have wild-type RAS genes (Yaeger et al., 2015). At present, the clinical treatment of mCRC is mainly based on radiotherapy, chemotherapy, and combination targeted therapy (Glynne-Jones et al., 2017; Modest et al., 2019; You et al., 2020). As a macromolecular monoclonal antibody–targeted drug, aflibercept can inhibit the growth, invasion, and metastasis of cancer cells by blocking the vascular endothelial growth factor (VEGF) (Holash et al., 2002; Saif, 2013). Based on the results of the randomized controlled trial VELOUR, the drug was approved by the FDA in 2012 in combination with FOLFIRI for second-line treatment of mCRC (FDA approves aflibercept, 2012; Li et al., 2018). Later, the therapy was approved in Japan and the European Union. On 13 February 2018, Bayer announced that the China Food and Drug Administration has approved the marketing application of aflibercept intraocular injection solution for the treatment of adult diabetic macular edema (DME) (Zhou et al., 2022). However, in mainland China, there is no aflibercept preparation for mCRC on the market; in other words, in mainland China, it has not yet been used for mCRC treatment. Situation differs in Hong Kong, Macau, and Taiwan regions of China, where aflibercept has already got approval for mCRC treatment in 2013–2014 (Yaozhi Data, 2014; Pharnexcloud, 2021; SSM, 2021).

Health technology assessment (HTA) can systematically evaluate the technical characteristics, effectiveness, safety, and socioeconomic attributes of health technologies, providing decision makers of health and healthcare and medical personnel with scientific information and an evidence-based basis for the rational choice of health technologies (Chen et al., 2018). It takes a lot of time and resources to carry out a comprehensive health technology assessment; when time and conditions are limited, through rapid assessment, the existing main evidence is sorted out and analyzed relatively efficiently, which can provide certain information support for decision makers in the clinical environment. The therapy has been approved for nearly a decade, and data on its efficacy, safety, and cost-effectiveness have been accumulating through this time. A certain number of secondary literature and economic studies on clinical efficacy and safety have been accumulated around aflibercept, which provide an evidence basis for rapid evaluation. The objective of this study is to evaluate the efficacy, safety, and cost-effectiveness of aflibercept in the treatment of mCRC in order to provide a decision-making reference for the selection of targeted drugs for second-line treatment of mCRC in Hong Kong, Macao, and Taiwan regions of China and the selection of new drugs for medical institutions in these regions.

## 2 Materials and methods

### 2.1 Inclusion and exclusion criteria

#### 2.1.1 Types of research

We included published HTA reports, systematic reviews (SR) or meta-analyses, and pharmacoeconomic studies.

#### 2.1.2 Research subjects

Patients diagnosed with mCRC were of any gender, ethnicity, onset, and origin, but all the patients should be adults. Considering the current status of antineoplastic drug research and the clinical characteristics of adverse events, for safety, a wider range of tumor patients were included for a more comprehensive assessment.

#### 2.1.3 Interventions

The trial group consisted of aflibercept monotherapy or chemotherapy (CT), and the control group was CT with or without other positive controls or the best supportive care. Both the experimental group and the control group were second-line treatments for mCRC, with unlimited doses and courses of treatments.

#### 2.1.4 Outcome indicators

Efficacy measures include overall survival (OS), progression-free survival (PFS), objective response rate (ORR), complete response (CR), partial response (PR), and disease control rate (DCR). Safety indicators include the incidence of overall adverse events (AE), incidence of serious adverse events, and incidence of various types of adverse events. Economic indicator includes the incremental cost-effectiveness ratio (ICER).

#### 2.1.5 Exclusion criteria

The exclusion criteria include (1) repeated publications, (2) literature with lack of data or inability to obtain the full text, and (3) non-Chinese and English literature.

### 2.2 Search strategy

We searched databases including PubMed, Embase, the Cochrane Library, CNKI, Wanfang, and Weipu, as well as the official websites and related databases including the National Institute of Health and Clinical Optimization, Centre for Evaluation and Communication at the University of York, and Canadian Agency for Medicines and Health Technology, and included HTA reports. SR or meta-analysis and pharmacoeconomic studies on aflibercept for metastatic colorectal cancer were searched in full text with *aflibercept*, *systematic review*, *Meta-analysis*, *economics*, *cost*, *economics*, and *health technology assessment* as keywords in English and Chinese, respectively, with a search time frame from the date of database creation to 11 November 2021. In addition, as a supplement, a manual search of references of included studies was conducted. The search strategy for PubMed, as an example, was as Table 1. The search strategies for other databases can be found in Supplementary Table S1.

**TABLE 1:** Search strategy for PubMed.

Database	PubMed
Search strategy	((aflibercept [mh]) OR aflibercept OR (VEGF Trap-regeneron) OR VEGF-Trap OR (VEGF Trap) OR (VEGF Trap-Eye) OR eylea OR Zaltrap OR (AVE 0005) OR AVE-0005 OR (AVE 005) OR AVE-005 OR AVE0005 OR AVE005 OR ZIV-aflibercept) AND ((systematic review) OR (meta) OR (economics) OR (cost) OR (health technology assessment))

### 2.3 Literature screening

After the literature was deduplicated, two researchers (Pu Ge and Xiaonan Wang) screened and cross-checked by reading the title, abstract, and full text according to the inclusion and exclusion criteria, and if there was any disagreement, they would negotiate with the third researcher (Xiao Han).

### 2.4 Literature extraction

The basic data were independently extracted by two researchers according to the pre-designed data extraction table, including first author, publication year, intervention/control measures, and outcome indicators. If the included literature was incomplete, we would contact the original author to obtain it.

### 2.5 Literature quality evaluation

Here, two investigators used different tools to evaluate the quality of all included literature. For HTA, the HTA checklist (Hailey and Topfer, 2003) was used for quality evaluation, which was an initiative of the International Network of Agencies for Health Technology Assessment (INAHTA); for systematic reviews/meta-analyses, the AMSTAR-2 scale (Shea et al., 2017) was used for quality evaluation; and for economic studies, the CHEERS scale (Husereau et al., 2013) was used for quality evaluation.

### 2.6 Data analysis

Because of the high heterogeneity of study types, this study used descriptive methods to assess outcomes. Qualitative profiling methods were used to classify, compare, and analyze the results of the included studies according to the study design, patient population, and intervention/control measures of each study.

## 3 Result

### 3.1 Literature search results

**FIGURE 1:**
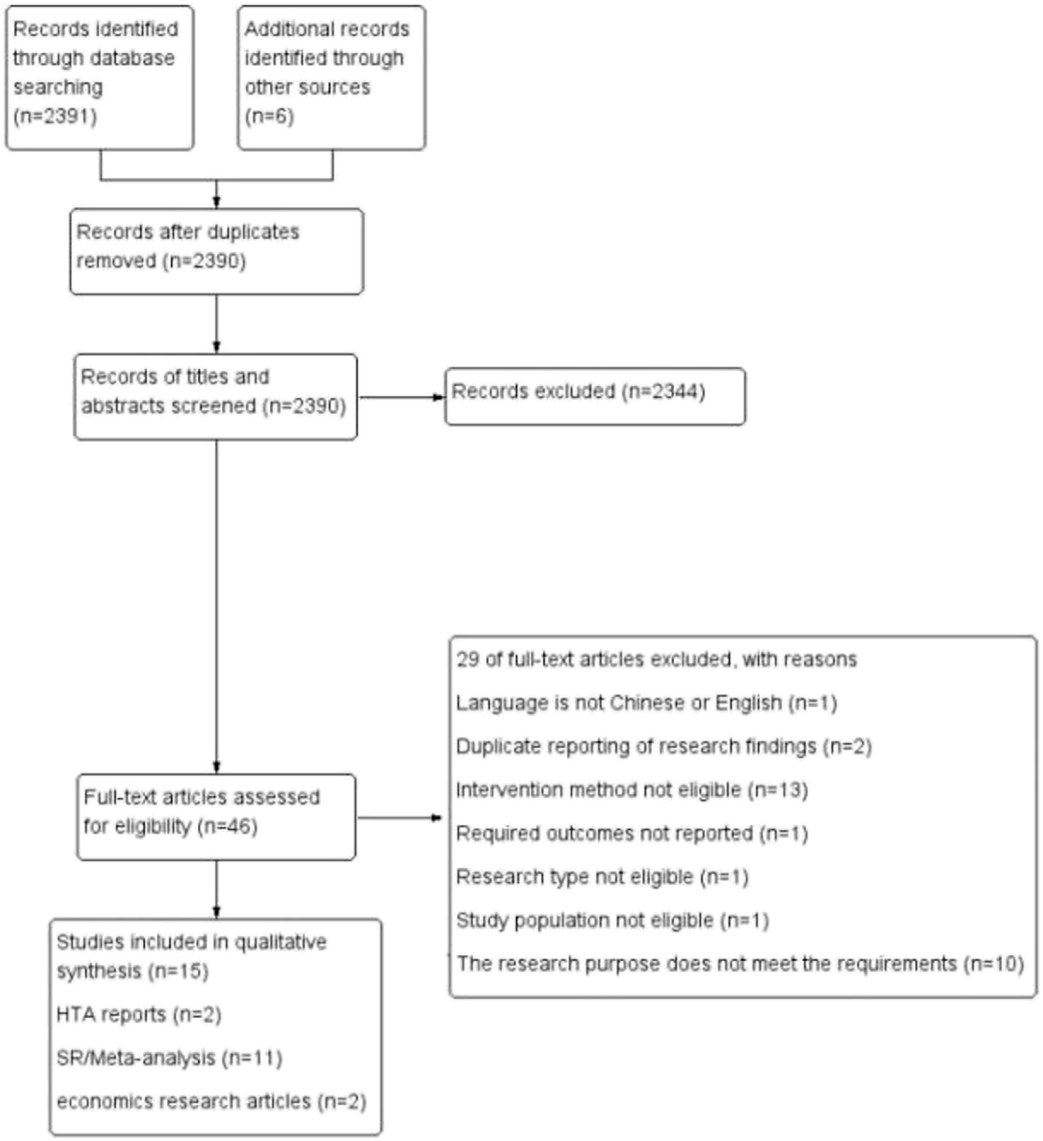
Flow chart of literature screening.

Figure 1 shows the flow chart of study selection. In the initial search, 2397 relevant research works were identified (290 research works from PubMed, 1055 from EMBASE, 149 from Cochrane Library, 877 from CNKI, 15 from Wanfang, five from Weipu, one from Center for Reviews and Dissemination of York University, and two from National Institute for Health Research of United Kingdom). After the exclusion of seven duplicate studies, 2390 studies underwent a title and abstract review. A total of 2344 studies were excluded at the initial screening stage. For the remaining 46 studies, the full text was reviewed, and 29 of them were excluded for following reasons: language is not Chinese or English (n = 1), duplication of research findings (n = 2), intervention method not eligible (n = 13), required outcomes not reported (n = 1), research type not eligible (n = 1), study population not eligible (n = 1), and research purpose fail to meet the requirements (n = 10). The 15 remaining studies fulfilled the eligibility criteria and were included in the rapid assessment, including two HTA reports, 11 SR/meta-analysis, and two cost-effectiveness research works.

### 3.2 Main characteristics and quality evaluation of the included literature

The main characteristics of the included HTA reports, SR/meta-analyses, and economics studies are reported in Tables 2-4. The overall quality of the literature was moderate for HTA reports, low for SR/meta-analyses, and high for economics studies.

**TABLE 2:** Basic characteristics of included HTAs.

Country	Institution	Evaluation time	Study population	Intervention measure	Control measure
Austrian (Rothschedl et al., 2013)	Austrian Ludwig Boltzmann Institute of Health Technology Assessment	2013	mCRC patients receiving second-line treatment	Aflibercept + FOLFIRI	FOLFIRI
United Kingdom (Wade et al., 2013)	University of York CRD and CHE Health technology assessment panel	2013	Patients with mCRC who are resistant to oxaliplatin-containing regimens or who have progressed after receiving the regimens described above	Aflibercept + FOLFIRI	FOLFIRI

**TABLE 3:** Data extraction of included SRs/meta-analyses.

Author, year	Research type	Retrieval time	Study population	No. (cases)	Total cases	Comparison group	Control group	Efficacy outcome index	Safety outcome index
(Kirstein et al., 2014)	Systematic review	2000–2014	mCRC patients	40	Not reported	Targeted drug (VEGFi and EGFRi) single agent or combination chemotherapy	Placebo + chemotherapeutic or chemotherapeutic only	Progression-free survival (PFS), overall survival (OS), and objective response rate (ORR)	Incidence of adverse events
(Gill et al., 2014)	Systematic review	2014.9.18	mCRC patients receiving non-first-line treatment	14	Not reported	Targeted drug (VEGFi and EGFRi) single agent or combination chemotherapy	Placebo + chemotherapeutic or chemotherapeutic only	Progression-free survival (PFS), overall survival (OS), and objective response rate (ORR)	Quality of life and incidence of adverse reactions
(Xu et al., 2021)	Mesh meta-analysis	2020.4.1	mCRC patients receiving second-line treatment	10	4183	Targeted drug (VEGFi and EGFRi) single agent or combination chemotherapy	Placebo + chemotherapeutic or chemotherapeutic only	Progression-free survival (PFS), overall survival (OS), and disease control rate (DCR)	None
(Xie et al., 2020)	Systematic review + mesh meta-analysis	2019.2	mCRC patients receiving second-line treatment	12	6805	Regorafenib in combination with chemotherapy	Bevacizumab, panitumumab, cetuximab, ramucirumab, canaklimumab, ganitumab (+nimotuzumab), and aflibercept in combination with chemotherapy	Progression-free survival (PFS), overall survival (OS), and disease control rate (DCR)	Incidence of grade ≥3 adverse reactions, incidence of neutropenia, incidence of febrile neutropenia, incidence of fatigue, and incidence of diarrhea
(Peng et al., 2014a)	Meta-analysis	2014.3	Patients with solid tumors	13	4538	Aflibercept alone or in combination with chemotherapy	Placebo + chemotherapeutic or chemotherapeutic only	None	Incidence of bleeding events of all grades and incidence of ≥ grade 3 (high-grade) bleeding events
(Peng et al., 2014b)	Meta-analysis	2014.3	Patients with solid tumors	16	4596	Aflibercept alone or in combination with chemotherapy	Placebo + chemotherapeutic or chemotherapeutic only	None	All-grade proteinuria event rate and ≥ grade 3 (high-grade) proteinuria event rate
(Zhang et al., 2016)	Systematic review + meta-analysis	2000.1–2015.3	Patients with solid tumors	10	4310	Aflibercept alone or in combination with chemotherapy	Placebo + chemotherapeutic or chemotherapeutic only	None	Incidence of unspecified infections, febrile neutropenia, sepsis, urinary tract infections, pneumonia, and fatal infections
(Qi et al., 2014a)	Systematic review + meta-analysis	2000–2014.1	Patients with solid tumors	8	4101	Aflibercept alone or in combination with chemotherapy	Placebo + chemotherapeutic or chemotherapeutic only	None	Incidence of gastrointestinal perforation events of all grades and incidence of ≥ grade 3 (high-grade) gastrointestinal perforation events
(Qi et al., 2014b)	Systematic review + meta-analysis	2013.8	Patients with solid tumors	15	4451	Aflibercept alone or in combination with chemotherapy	Placebo + chemotherapeutic or chemotherapeutic only	None	Incidence of hypertensive events of all grades and ≥ grade 3 (high-grade) hypertensive events
(Kanukula et al., 2019)	Systematic review + meta-analysis	2010.1–2017.9	Patients with solid tumors	7	4389	Aflibercept alone or in combination with chemotherapy	Placebo + chemotherapeutic or chemotherapeutic only	None	Incidence of all grades of venous thromboembolic events and incidence of ≥ grade 3 (high-grade) venous thromboembolic events
(Qi et al., 2014c)	Meta-analysis	2000–2013.8	Patients with solid tumors	10	3060	Aflibercept alone or in combination with chemotherapy	Placebo + chemotherapeutic or chemotherapeutic only	None	Treatment-related mortality

**TABLE 4:** Summary of included pharmacoeconomic studies.

Author, years	Location	View	Economic research methods	Economic model	Time limit	Disease	Intervention measure	Control measures
(Wade et al., 2015)	United Kingdom.	Third party payer	Cost-effectiveness	Three-state Markov model	15 years	mCRC patients receiving second-line therapy	Aflibercept + FOLFIRI	FOLFIRI
(Kashiwa and Matsushita, 2020)	Japan	Healthcare payer	Cost-effectiveness	Partition survival model	10 years	mCRC patients receiving second-line therapy	Aflibercept + FOLFIRI	#2C2F34 Ramucirumab + FOLFIRI and FOLFIRI

### 3.3 Quality evaluation of included studies

#### 3.3.1 Quality evaluation of HTA reports

The quality evaluation results of HTA are shown in Table 5. It can be seen from Table 4 that the HTA reports included in this study are of high quality.

**TABLE 5:** Quality evaluation of included HTA.

		Authors	
		Eleen R (Rothschedl et al., 2013)	Wade R (Wade et al., 2013)
Items	1 Appropriate contact details for further information?	Yes	Yes
	2 Authors identified?	Yes	Yes
	3 Statement regarding conflict of interest?	No	Yes
	4 Statement on whether report was externally reviewed?	Yes	Yes
	5 Short summary in non-technical language?	Partial Yes	Partial Yes
	6 Reference to the policy question that is addressed?	No	Partial Yes
	7 Reference to the research question(s) that is/are addressed?	Partial Yes	Yes
	8 Scope of the assessment specified?	Yes	Yes
	9 Description of the assessed health technology?	Yes	Yes
	10 Details on sources of information and literature search strategies were provided?		
	10.1 Search strategy	Yes	Yes
	10.2 Databases	Yes	Yes
	10.3 Year range	Yes	Yes
	10.4 Language restriction	No	Yes
	10.5 Primary data	Partial Yes	Yes
	10.6 Other kinds of information resources	Yes	Yes
	10.7 Complete reference list of included studies	Yes	Yes
	10.8 List of excluded studies	No	No
	10.9 Inclusion criteria	Partial Yes	Yes
	10.10 Exclusion criteria	No	Yes
	11 Information on the basis for the assessment and interpretation of selected data and information?		
	11.1 Method of data extraction described?	No	Yes
	11.2 Critical appraisal method (for quality assessment of the literature) described?	No	Partial Yes
	11.3 Method of data synthesis described?	No	Yes
	11.4 Results of the assessment clearly presented, for example, in the form of evidence tables?	Yes	Yes
	12 (Medico-) legal implications considered?	No	No
	13 Economic analysis provided?	Yes	Yes
	14 Ethical implications considered?	No	No
	15 Social implications considered?	No	No
	16 Other perspectives (stakeholders, patients, and consumers) considered?	No	No
	17 Findings of the assessment discussed?	Yes	Yes
	18 Conclusions from assessment clearly stated?	Partial Yes	Yes
	19 Suggestions for further action?	Partial Yes	Partial Yes

#### 3.3.2 Quality evaluation of SR/meta-analysis

The quality evaluation results of the SR/Meta-analysis are shown in Table 6. Table 7 shows that the quality of the SR/meta-analysis included in this study is relatively low. Studies excluding Xiaoyu Xie (Peng et al., 2014a; Qi et al., 2014a; Peng et al., 2014b; Qi et al., 2014b; Qi et al., 2014c; Gill et al., 2014; Kirstein et al., 2014; Zhang et al., 2016; Kanukula et al., 2019; Xie et al., 2020; Xu et al., 2021) had defects in “Item 2”. This item required the author to write a research plan and register or publish it before conducting a systematic review, but none of the studies mentions the existence of the plan. “Item seven” required a list of excluded studies and reasons for exclusion, but none of the included studies (Peng et al., 2014a; Qi et al., 2014a; Peng et al., 2014b; Qi et al., 2014b; Qi et al., 2014c; Gill et al., 2014; Kirstein et al., 2014; Zhang et al., 2016; Kanukula et al., 2019; Xie et al., 2020; Xu et al., 2021) provided a detailed list of excluded studies. The aforementioned two items were important areas of the quality evaluation, so the quality evaluation results of SR/meta-analysis were relatively low.

**TABLE 6:** Quality evaluation of included SR/meta-analysis.

		Author(s)										
		Martha M. Kirstein (Kirstein et al., 2014)	Sharlene Gill (Gill et al., 2014)	Zhili Xu (Xu et al., 2021)	Xiaoyu Xie (Xie et al., 2020)	Ling Peng 1 (Peng et al., 2014a)	Ling Peng 2 (Peng et al., 2014b)	Xi Zhang (Zhang et al., 2016)	Wei-Xiang Qi 1 (Qi et al., 2014a)	Wei-Xiang Qi 2 (Qi et al., 2014b)	Raju Kanukula (Kanukula et al., 2019)	Wei-Xiang Qi 3 (Qi et al., 2014c)
Items	1 Did the research questions and inclusion criteria for the review include the components of PICO?	No	No	Yes	Yes	Yes	Yes	Yes	Yes	Yes	No	Yes
	2 Did the report of the review contain an explicit statement that the review methods were established prior to the conduction of the review and did the report justify any significant deviations from the protocol?	No	No	No	Yes	No	No	No	No	No	No	No
	3 Did the review authors explain their selection of the study designs for inclusion in the review?	No	No	No	No	Yes	Yes	Yes	Yes	No	Yes	Yes
	4 Did the review authors use a comprehensive literature search strategy?	Partial Yes	Partial Yes	Partial Yes	Partial Yes	Partial Yes	Partial Yes	Partial Yes	Partial Yes	Partial Yes	Partial Yes	Partial Yes
	5 Did the review authors perform study selection in duplicate?	Yes	No	No	No	Yes	Yes	Yes	No	Yes	Yes	No
	6 Did the review authors perform data extraction in duplicate?	No	No	Yes	Yes	No	No	Yes	Yes	No	Yes	Yes
	7 Did the review authors provide a list of excluded studies and justify the exclusions?	No	No	No	No	No	No	No	No	No	No	No
	8 Did the review authors describe the included studies in adequate detail?	Partial Yes	Partial Yes	Partial Yes	Partial Yes	Partial Yes	Partial Yes	Partial Yes	Partial Yes	Partial Yes	No	Partial Yes
	9.1 Did the review authors use a satisfactory technique for assessing the risk of bias (RoB) in individual studies that were included in the review?(RCT)	No	No	Yes	Yes	No	No	No	No	No	Yes	No
	9.2 Did the review authors use a satisfactory technique for assessing the risk of bias (RoB) in individual studies that were included in the review? (Non-RCT)	Yes	Yes	Yes	Yes	Yes	Yes	Yes	Yes	Yes	Yes	Yes
	10 Did the review authors report on the sources of funding for the studies included in the review?	No	No	No	No	No	No	No	No	No	No	No
	11 If meta-analysis was performed did the review authors use appropriate methods for the statistical combination of results?	No meta-analysis conducted	No meta-analysis conducted	Yes	Yes	Yes	Yes	Yes	Yes	Yes	Yes	Yes
	12 If meta-analysis was performed, did the review authors assess the potential impact of RoB in individual studies on the results of the meta-analysis or other evidence synthesis?	No meta-analysis conducted	No meta-analysis conducted	No	No	No	No	No	No	No	No	No
	13 Did the review authors account for RoB in individual studies when interpreting/discussing the results of the review?	No	No	Yes	Yes	Yes	Yes	Yes	Yes	Yes	Yes	Yes
	14 Did the review authors provide a satisfactory explanation for, and discussion of, any heterogeneity observed in the results of the review?	No	No	Yes	Yes	Yes	Yes	Yes	Yes	Yes	No	Yes
	15 If they performed quantitative synthesis did the review authors carry out an adequate investigation of publication bias (small study bias) and discuss its likely impact on the results?	No meta-analysis conducted	No meta-analysis conducted	Yes	Yes	Yes	Yes	Yes	Yes	Yes	No	No
	16 Did the review authors report any potential sources of conflict of interest, including any funding they received for conducting the review?	Yes	Yes	Yes	Yes	Yes	Yes	Yes	Yes	Yes	Yes	Yes
Grades		Very low	Very low	Very low	Low	Very low	Very low	Very low	Very low	Very low	Very low	Very low

**TABLE 7:** Quality evaluation of included pharmacoeconomic studies.

		Author(s)	
		Ros Wade (Wade et al., 2015)	Munenobu Kashiwa (Kashiwa and Matsushita, 2020)
Items	1 Title	Yes	Yes
	2 Abstract	Partial Yes	Yes
	3 Background and objectives	Yes	Yes
	4 Target population and subgroups	Yes	Yes
	5 Setting and location	Yes	Yes
	6 Study perspective	Yes	Yes
	7 Comparators	Yes	Yes
	8 Time horizon	Yes	Yes
	9 Discount rate	Yes	Yes
	10 Choice of health outcomes	Yes	Yes
	11 Measurement of effectiveness	Yes	Yes
	12 Measurement and valuation of preference-based outcomes	Partial Yes	Partial Yes
	13 Estimating resources and costs	Yes	Yes
	14 Currency, price date, and conversion	Yes	Yes
	15 Choice of model	Yes	Yes
	16 Assumptions	Partial Yes	Yes
	17 Analytical methods	Partial Yes	Partial Yes
	18 Study parameters	Partial Yes	Yes
	19 Incremental costs and outcomes	Yes	Yes
	20 Characterizing uncertainty	Partial Yes	Yes
	21 Characterizing heterogeneity	Yes	No
	22 Study findings, limitations, generalizability, and current knowledge	Yes	Yes
	23 Source of funding	Yes	No
	24 Conflicts of interest	Yes	Yes

#### 3.3.3 Quality evaluation of economic research

The quality evaluation results of economic research are shown in Table 7. According to Table 6, in Wade et al. (2015), the complete coincidence rate of each item on the CHEERS scale was 75% and the total coincidence rate was 100%, while in Kashiwa and Matsushita (2020), the complete coincidence rate of each item on the CHEERS scale was 83.33% and the total coincidence rate was 91.67%, both of which were relatively high. The quality of economic research included was relatively high.

### 3.4 Effectiveness evaluation

#### 3.4.1 Evaluation of the effectiveness of aflibercept monotherapy in mCRC

The HTA report (Rothschedl et al., 2013) from the Ludwig Boltzmann Institute for Health Technology Assessment in Austria reported the results of a phase II clinical trial of aflibercept monotherapy in metastatic colorectal cancer (Tang et al., 2012). The study was conducted at seven Canadian clinical research centers and one American research center. In this study, patients were divided into two arms according to whether they had received bevacizumab before: the arm that had not received bevacizumab before (arm 1) (n = 24) and the arm that had received bevacizumab before (arm 2) (n = 50). Among the 24 patients who had not received bevacizumab before, eight (33.33%) patients had stable disease for 8 weeks, and five (20.83%) patients had stable disease for over 16 weeks. Median PFS was 2 months (95% CI 1.7–8.6 months) for the arm not received bevacizumab before and 2.4 months (95% CI 1.9–3.7 months) for the arm that received bevacizumab before, respectively. Also, median OS was 10.4 months (95% CI 7.6–15.5 months) and 8.5 months (95% CI 6.2–10.6 months), respectively.

#### 3.4.2 Evaluation of the effectiveness of aflibercept combined with FOLFIRI in mCRC

A total of two HTA reports (Rothschedl et al., 2013; Wade et al., 2013) and four SR/meta-analyses (Gill et al., 2014; Kirstein et al., 2014; Xie et al., 2020; Xu et al., 2021) reported the effectiveness of aflibercept in combination with FOLFIRI for mCRC. Among them, there were two HTA reports and three SR/meta-analyses (Rothschedl et al., 2013; Wade et al., 2013; Gill et al., 2014; Kirstein et al., 2014; Xie et al., 2020) that included VELOUR (a randomized controlled trial), and one SR/meta-analysis (Xu et al., 2021) included VELOUR and another randomized controlled trial.

VELOUR (Qi et al., 2014b) is a prospective, multinational, randomized, double-blind, parallel phase III randomized clinical study conducted in 176 clinical research centers in 28 countries. In this study, patients with metastatic colorectal cancer previously treated with oxaliplatin-based chemotherapy regimens were randomized into two arms: the experimental group (n = 612) treated with aflibercept plus FOLFIRI and the control group (n = 614) treated with placebo plus FOLFIRI. The objective response rate was 19.8% in the experimental group and 11.1% in the control group. Results show a significant difference between the experimental group and the control group (*p* < 0.001). The median PFS of the experimental group was 6.90 months (95% CI 6.51–7.2 months) and that of the control group was 4.67 months (95% CI 4.21–5.36 months); the HR of the experimental group to the control group was 0.758 (95% CI 0.661–0.869, *p* < 0.0001). The median OS of the experimental group was 13.50 months (95% CI 12.517–14.949 months) and that of the control group was 12.06 months (95% CI 11.072–13.109 months); the HR of the experimental group to the control group was 0.817 (95% CI 0.713–0.937, *p* = 0.0032). There were significant differences in median PFS and median OS between the experimental and control groups. The study also found that the efficacy of aflibercept was not related to previous bevacizumab treatment. This study provides crucial evidence for the approval of aflibercept combined with FOLFIRI for second-line treatment in patients with metastatic colorectal cancer who progressed or were resistant to oxaliplatin after oxaliplatin treatment.

Xu et al. (2021) conducted a network meta-analysis to compare the efficacy of second-line treatment for metastatic colorectal cancer. In total, two randomized controlled trials with aflibercept were included in the study. The experimental group was aflibercept plus chemotherapy, and the control group was placebo plus chemotherapy. In these two trials, compared with the control group, the HR of OS was 0.81 (95% CI 0.72–0.92) and that of PFS was 0.69 (95% CI 0.54–0.88). At the same time, the researchers compared the OS and PFS of the aflibercept plus chemotherapy group with other second-line therapies, such as bevacizumab plus chemotherapy, cetuximab plus chemotherapy, ramucirumab plus chemotherapy, and panitumumab plus chemotherapy, but no significant difference was found.

Xie et al. (2020) conducted a mesh meta-analysis to compare the efficacy of regorafenib plus chemotherapy with other second-line regimens for metastatic colorectal cancer. In this study, aflibercept plus chemotherapy and regorafenib plus chemotherapy were indirectly compared, with HR 0.81 for OS (95% CI 0.55–1.18), 1.04 for PFS (95% CI 0.73–1.47), and OR 0.988 for ORR (95% CI 0.413–2.18). There was no significant difference in the efficacy between aflibercept plus chemotherapy and regorafenib plus chemotherapy in this study.

### 3.5 Safety evaluation

In total, two HTA and nine SR/meta-analyses (Rothschedl et al., 2013; Wade et al., 2013; Peng et al., 2014a; Qi et al., 2014a; Peng et al., 2014b; Qi et al., 2014b; Qi et al., 2014c; Gill et al., 2014; Kirstein et al., 2014; Zhang et al., 2016; Kanukula et al., 2019) reported the safety of the VELOUR test. In the VELOUR test (Van Cutsem et al., 2012), the incidence of all-grade adverse events in the experimental group (aflibercept plus FOLFIRI) was 99.2%, grade 3 was 62.0%, and grade 4 was 21.4%. In the control group (placebo plus FOLFIRI), the incidence of all-grade adverse events was 97.9%, grade 3 was 45.1%, and grade 4 was 17.4%. The incidence of anti-VEGF–related adverse events (hypertension, hemorrhagic, thromboembolism, and proteinuria) and chemotherapy-related adverse events (diarrhea, weakness, stomatitis, palmar-plantar erythrodysesthesia syndrome, grade 3/4 neutropenia, and thrombocytopenia) in the experimental group was also higher than those in the control group.

A total of seven meta-analyses examined the incidence of different adverse events in patients with solid tumors treated with aflibercept. The results of six meta-analyses showed that patients with solid tumors treated with aflibercept had a higher rate of all-grade (RR = 2.63, 95% CI 2.07–3.34) and high-grade hemorrhagic events (RR = 2.45, 95% CI 1.62–3.72), all-grade (RR = 1.41, 95% CI 1.13–1.77) and high-grade proteinuria (RR = 6.79, 95% CI 3.10–14.89), high-grade (RR = 1.87, 95% CI 1.52–2.30) and fatal infection (OR = 2.16, 95% CI 1.14–4.11), all-grade (OR = 3.76, 95% CI 1.94–7.25) and high-grade gastrointestinal perforation (OR = 4.14, 95% CI 2.12–8.06), all-grade (OR = 4.47, 95% CI 3.84–5.22) and high-grade hypertension (OR = 4.97, 95% CI 3.95–6.27), and the risk of treatment-related death (OR = 1.81, 95% CI 1.20–2.72) than those in the control group which was only treated with chemotherapy. In contrast, the results of a meta-analysis (Peng et al., 2014b) showed that in the mCRC population, there was no significant difference in the risk of all-grade (RR = 1.00 95% CI 0.67–1.51) and high-grade venous thromboembolism (RR = 1.08 95% CI 0.67–1.73) in the experimental group (with aflibercept) compared to the control group.

### 3.6 Economic evaluation

In total, two pharmacoeconomic studies (Wade et al., 2015; Kashiwa and Matsushita, 2020) included cost-effectiveness analysis: one conducted in the United Kingdom and the other in Japan. Qi et al. (2014a) compared the cost-effectiveness of aflibercept combined with FOLFIRI and FOLFIRI alone. Kashiwa and Matsushita (2020) compared the cost-effectiveness of aflibercept combined with FOLFIRI, ramucirumab combined with FOLFIRI, and FOLFIRI alone.

Wade et al. (2015) described the manufacturer’s cost-effectiveness analysis of aflibercept FOLFIRI for second-line treatment of mCRC and the review of the manufacturer’s cost-effectiveness analysis results by the Evidence Review Group (ERG). The manufacturer used the three-state Markov model to simulate the cost-effectiveness after 15 years of treatment with aflibercept plus FOLFIRI. The cost included drug cost, adverse event treatment cost, and follow-up treatment cost. The manufacturer’s cost-effectiveness results showed that the ICER per QALY (quality-adjusted life year) of aflibercept combined with FOLFIRI is £ 36,294/QALY compared with FOLFIRI. The ERG believed that the manufacturer’s estimate of the efficacy of aflibercept + FOLFIRI was too optimistic. The manufacturer did not fully consider the cost of drug infusion and management. In addition, the patient group in the manufacturer’s model was younger than the actual population. After the preliminary review by the ERG, the manufacturer revised the model to include an additional cost of £ 15 for the infusion of aflibercept and £ 45 for additional dosing time and re-estimated the efficacy of aflibercept + FOLFIRI. In the revised model, compared with FOLFIRI, the ICER of aflibercept combined with FOLFIRI was £ 42,242/QALY. The ERG believed that the ICER estimation of each QALY of aflibercept combined with FOLFIRI in the revised model was still too low and did not correspond to the actual situation. The ERG estimated that the ICER of aflibercept combined with FOLFIRI was between £ 50,991/QALY and £ 55,139/QALY compared with FOLFIRI. Based on the cost-effectiveness analysis results provided by the manufacturer and the analysis of the evidence review team, the evidence review team believed that aflibercept combined with FOLFIRI had no cost-effectiveness advantage in the treatment of MCRC compared with FOLFIRI. Finally, NICE issued guidance based on the available findings that abciximab in combination with FOLFIRI was not recommended for the treatment of mCRC progressed or was resistant to oxaliplatin after oxaliplatin treatment.

Kashiwa and Matsushita (2020), based on the perspective of medical insurance payers, using partitioned survival analysis, simulated the cost-effectiveness of mCRC patients after 10 years of treatment with aflibercept plus FOLFIRI, ramucirumab plus FOLFIRI, or FOLFIRI treatment. Drug costs (targeted drugs, chemotherapy drugs, and other drugs) and other costs (various testing costs, contrast agent costs, prescription costs, dispensing costs, follow-up costs, and chemotherapy management costs) were considered. The analysis showed that in Japan, the combination of aflibercept or ramucirumab with FOLFIRI was not cost-effective compared with FOLFIRI treatment, although it could improve the efficacy. Compared with FOLFIRI, the ICER of aflibercept or ramucirumab combined with FOLFIRI was $31010/QALY and $52229/QALY, respectively. It was more cost-effective to add aflibercept than to add ramucirumab.

## 4 Discussion

According to the rapid health technology assessment, for mCRC patients who were resistant or progressing after first-line oxaliplatin treatment, aflibercept combined with FOLFIRI could improve PFS, OS, and ORR compared with FOLFIRI alone. Somehow, a clinical study (Tang et al., 2012) included in the HTA from Austria showed that aflibercept as a single agent for the second-line treatment of mCRC had no significant therapeutic effect. Furthermore, two studies of network meta-analysis (Xie et al., 2020; Xu et al., 2021) compared the efficacy of aflibercept plus FOLFIRI with other second-line therapies (regorafenib plus FOLFIRI, bevacizumab plus chemotherapy, cetuximab plus chemotherapy, ramucirumab plus chemotherapy, and panitumumab plus chemotherapy) in the mCRC treatment, but no significant difference in efficacy was found among aflibercept plus FOLFIRI and other included second-line therapies.

However, compared with FOLFIRI, aflibercept plus FOLFIRI had a higher incidence of high-grade adverse events in mCRC treatment (Van Cutsem et al., 2012; Peng et al., 2014a; Qi et al., 2014a; Peng et al., 2014b; Qi et al., 2014b; Qi et al., 2014c; Zhang et al., 2016). Adverse events in patients treated with aflibercept plus FOLFIRI were divided into two categories: one was anti-VEGF–related adverse events, such as hypertension, hemorrhagic events, thromboembolism, and proteinuria, and the other was chemotherapy-related adverse events, such as diarrhea, weakness, stomatitis, hand-foot syndrome, neutropenia, and thrombocytopenia. The application of aflibercept increased the incidence of these two categories of adverse events, which caused many patients to stop treatment, and a large number of patients had to bear additional adverse reaction management costs for this, which would also increase their financial burden. Several studies (Peng et al., 2014a; Peng et al., 2014b; Gill et al., 2014; Zhang et al., 2016) compared the risk of adverse events in patients with cancer treated with aflibercept or bevacizumab and found that the overall risk of high-grade adverse events in patients receiving aflibercept was higher than that of patients receiving bevacizumab. The risks of several types of adverse events such as high-grade bleeding, proteinuria, and infections in patients receiving aflibercept were also significantly higher than those in patients receiving bevacizumab.

In total, two cost-effectiveness analysis studies (Wade et al., 2015; Kashiwa and Matsushita, 2020) showed that, compared with FOLFIRI, aflibercept combined with FOLFIRI had higher ICER in mCRC patients in the United Kingdom and Japan, and the ICER was also higher than the willingness to pay threshold of their country. Manufacturers should further improve the production process, and relevant agencies in various countries should actively negotiate with manufacturers to work together to reduce the price of the drug and increase its availability.

Among the clinical studies of aflibercept combined with chemotherapy in mCRC, only one RCT was conducted in several study sites including China (including mainland China, Hong Kong, and Taiwan), which is AFLAME by Li et al. (2018). This was a prospective, multicenter, multinational, randomized, double-blind, parallel group, phase III study carried out at 37 active sites in mainland China, Hong Kong, Japan, Singapore, and Taiwan. Patients aged 18 years or older with histologically or cytologically proven adenocarcinoma of the colon or rectum that was metastatic and not amenable to potentially curative treatment (i.e., inoperable) were eligible. Patients were randomly assigned (2:1) to aflibercept plus FOLFIRI or placebo plus FOLFIRI centrally *via* an interactive voice response system (IVRS) using permuted-block randomization, stratified according to baseline ECOG performance status (0 vs. 1) and prior bevacizumab (yes vs. no). Between 27 July 2012 and 19 March 2014, 332 patients were enrolled and randomly assigned to treatment groups: 223 to aflibercept plus FOLFIRI and 109 to placebo plus FOLFIRI. But in this clinical study, some problems arose. Due to the clinical supply misallocation, 198 (60%) of 332 patients received at least one cycle of misallocated treatment (aflibercept or placebo, all still received FOLFIRI): 122 of 223 in the aflibercept plus FOLFIRI group and 76 of 109 in the placebo plus FOLFIRI group. Finally, 111 patients received aflibercept plus FOLFIRI, 188 patients received mixed administration, and 33 patients received placebo plus FOLFIRI. The Data Monitoring Committee did not stop the study despite the misallocation. Ultimately, the researchers concluded that despite the misallocation, the study demonstrated that the addition of aflibercept to FOLFIRI chemotherapy improved PFS, overall survival, and response rate in patients from the Asia-Pacific region with oxaliplatin-pretreated mCRC. No new safety concerns were identified in this patient population. Together, these data suggest a favorable benefit–risk ratio for the aflibercept plus FOLFIRI combination in this setting. This study provides some evidence for the launch of aflibercept in combination with FOLFIRI in the treatment of mCRC in China, but due to the limitations of this study, the results of this study should be treated with caution.

At present, the guidelines for mCRC in France, the United States, and Japan (Aparicio et al., 2020; Hashiguchi et al., 2020; Benson et al., 2021) recommend that aflibercept can be combined with FOLFIRI for the second-line treatment of mCRC. However, the US NCCN guidelines (Benson et al., 2021) also pointed out that bevacizumab has a higher priority than aflibercept in the second-line treatment of mCRC, which was in contrast to the relatively higher price and relatively higher adverse effect rate of aflibercept. However, if the patient was resistant to bevacizumab, aflibercept was a viable option for second-line treatment. In clinical work, doctors should evaluate the health and physical strength of patients in various aspects, improve examinations, and select drugs based on the actual conditions of the patients.

This study has several advantages. It comprehensively summarizes the secondary evidence of aflibercept combined with chemotherapy for the treatment of mCRC and comprehensively analyzes the efficacy, safety, and cost-effectiveness of the therapy, which provides not only some evidence-based evidence for the clinical application of this therapy but also evidence for its drug selection decision in Hong Kong, Macao, and Taiwan regions of China.

This study also has some limitations. First, it focused on the analysis of the efficacy of aflibercept combined with FOLFIRI in the second-line treatment of mCRC. Due to the lack of direct head-to-head studies, the subgroup data of the network meta-analysis included was partially adopted. The quality of the evidence may be lower than direct research. The results might be affected by publication bias. Second, this study was a rapid assessment, with mainly qualitative analysis as well as potentially limited result.

## 5 Conclusion

Compared with FOLFIRI, aflibercept combined with FOLFIRI in the second-line mCRC treatment had better efficacy, but it was less safe and did not have a cost-effectiveness advantage. In the future, randomized controlled trials should be further carried out to clarify the effectiveness and safety of this therapy, and its effectiveness and safety should be compared with other second-line therapies such as bevacizumab, cetuximab, and regorfenib. Medical institutions in Hong Kong, Macao, and Taiwan regions of China should be cautious when using or introducing aflibercept plus FOLFIRI as mCRC treatment.

## Data Availability

The original contributions presented in the study are included in the article/Supplementary Material; further inquiries can be directed to the corresponding author.
