# Supplementary material for: Efficacy, safety, and cost-effectiveness analysis of aflibercept in metastatic colorectal cancer: A rapid health technology assessment
Source: Front Pharmacol. 2022 Aug 30;13:914683. doi: 10.3389/fphar.2022.914683 (PMC9469875; doi:10.3389/fphar.2022.914683)
Supplement: Supplementary file 1 [file Table1.DOCX]

| **Supplementary Table 1 Retrieval strategy of each database except pubmed** | |
| --- | --- |
| Search date | 9 September 2021 |
| 知网/CNKI | FT='系统评价'+'系统综述'+'荟萃分析'+'meta分析'+'成本'+'费用'+'经济学'+'卫生技术评估' AND FT=‘阿柏西普’ |
| 万方/Wangfang | 全部:“阿柏西普” and （全部:“系统评价” or 全部:“系统综述”or 全部:“荟萃分析”or 全部:“meta分析”or 全部:“成本”or 全部:“费用” or 全部:“经济学”or 全部:“卫生技术评估”） |
| 维普/Weipu | U=阿柏西普 AND （U=系统评价 OR 系统综述 OR 荟萃分析 OR meta分析 OR 成本 OR 费用 OR 卫生技术评估 OR 经济学） |
| Embase | ('aflibercept'/exp OR 'aflibercept' OR 'vegf trap-regeneron' OR 'vegf-trap' OR 'vegf trap' OR 'vegf trap-eye' OR 'eylea' OR 'zaltrap' OR 'ave 0005' OR 'ave-0005' OR 'ave 005' OR 'ave-005' OR 'ave0005' OR 'ave005' OR 'ziv-aflibercept') AND ('systematic review'/exp OR 'systematic review' OR 'meta' OR 'cost'/exp OR 'cost' OR 'economics'/exp OR 'economics' OR 'health technology assessment'/exp OR 'health technology assessment') |
| Cochrane | #1 (aflibercept)  #2 (VEGF Trap-regeneron)  #3 (VEGF-Trap)  #4 (VEGF Trap-Eye)  #5 (eylea)  #6 (Zaltrap)  #7 (AVE 0005)  #8 (AVE-0005)  #9 (AVE 005)  #10 (AVE-005)  #11 (AVE0005)  #12 (AVE005)  #13 (ZIV-aflibercept)  #14 (VEGF Trap)  #15 #1 OR #2 OR #3 OR #4 OR #5 OR #6 OR #7 OR #8 OR #9 OR #10 OR #11 OR #12 OR #13 OR #14  #16 systematic review  #17 meta  #18 economic  #19 Heath technology assessment  #20 cost  #21 #16 OR #17 OR #18 OR #19 OR #20  #22 #15 AND #21 |
